# The Relevance of a Novel Quantitative Assay to Detect up to 40 Major *Streptococcus pneumoniae* Serotypes Directly in Clinical Nasopharyngeal and Blood Specimens

**DOI:** 10.1371/journal.pone.0151428

**Published:** 2016-03-17

**Authors:** Melina Messaoudi, Milen Milenkov, Werner C. Albrich, Mark P. G. van der Linden, Thomas Bénet, Monidarin Chou, Mariam Sylla, Patricia Barreto Costa, Nathalie Richard, Keith P. Klugman, Hubert P. Endtz, Gláucia Paranhos-Baccalà, Jean-Noël Telles

**Affiliations:** 1 Emerging Pathogens Laboratory, Fondation Mérieux - Centre International de Recherche en Infectiologie (CIRI), Lyon, France; 2 Departement of Medical Microbiology & Infectious Diseases Erasmus MC, Rotterdam, The Netherlands; 3 Department of Infectious Diseases and Hospital Epidemiology, Kantonsspital St. Gallen, St. Gallen, Switzerland; 4 Medical Research Council Respiratory and Meningeal Pathogens Research Unit, University of the Witwatersrand, Johannesburg, South Africa; 5 National Reference Center for Streptococci, Institute of Medical Microbiology, University Hospital RWTH Aachen, Aachen, Germany; 6 Service d'Hygiène, Epidémiologie et Prévention, Hôpital Edouard Herriot - Hospices Civils de Lyon, France; 7 Faculty of Pharmacy, University of Health Sciences, Phnom Penh, Cambodia; 8 CHU Gabriel Touré, Bamako, Mali; 9 Laboratório de vírus respiratórios e do sarampo, Instituto Oswaldo Cruz/Fiocruz, Rio de Janeiro, Brazil; 10 Service de Réanimation Pédiatrique Médico-Chirurgicale, HFME, Groupement Hospitalier Est, Bron, France; 11 Hubert Department of Global Health and Division of Infectious Diseases, Emory University, Atlanta, Georgia, United States of America; Rockefeller University, UNITED STATES

## Abstract

For epidemiological and surveillance purposes, it is relevant to monitor the distribution and dynamics of *Streptococcus pneumoniae* serotypes. Conventional serotyping methods do not provide rapid or quantitative information on serotype loads. Quantitative serotyping may enable prediction of the invasiveness of a specific serotype compared to other serotypes carried. Here, we describe a novel, rapid multiplex real-time PCR assay for identification and quantification of the 40 most prevalent pneumococcal serotypes and the assay impacts in pneumonia specimens from emerging and developing countries. Eleven multiplex PCR to detect 40 serotypes or serogroups were optimized. Quantification was enabled by reference to standard dilutions of known bacterial load. Performance of the assay was evaluated to specifically type and quantify *S*. *pneumoniae* in nasopharyngeal and blood samples from adult and pediatric patients hospitalized with pneumonia (*n* = 664) from five different countries. Serogroup 6 was widely represented in nasopharyngeal specimens from all five cohorts. The most frequent serotypes in the French, South African, and Brazilian cohorts were 1 and 7A/F, 3 and 19F, and 14, respectively. When both samples were available, the serotype in blood was always present as carriage with other serotypes in the nasopharynx. Moreover, the ability of a serotype to invade the bloodstream may be linked to its nasopharyngeal load. The mean nasopharyngeal concentration of the serotypes that moved to the blood was 3 log-fold higher than the ones only found in the nasopharynx. This novel, rapid, quantitative assay may potentially predict some of the *S*. *pneumoniae* serotypes invasiveness and assessment of pneumococcal serotype distribution.

## Introduction

*Streptococcus pneumoniae* is the major pathogen implicated in community-acquired pneumonia (CAP) in adults and children. CAP is a major public health problem worldwide; more than one million people die of pneumococcal disease every year including ~800,000 deaths in children aged under five [1], mostly in developing countries [2,3]. *S*. *pneumoniae* is a frequent commensal of the upper respiratory tract in humans. Asymptomatic colonization rates vary from less than 5% in adults to more than 50% in young children [3–5].

More than 90 different serotypes of *S*. *pneumoniae* have been identified to date on the basis of the biochemical structure of the capsular polysaccharide. Considering that the capsule is a major virulence factor for pneumococci, serotype identification is important to study the relationship between capsule composition and the severity of infection. About 10 different *S*. *pneumoniae* serotypes have been described to account for the majority of invasive pneumococcal disease worldwide [6]. Prior to the introduction of pneumococcal vaccines, a limited number of serotypes and serogroups were frequently associated with pediatric disease (serotypes 4 and 14 and serogroups 6, 7, 9, 18, 19, and 23) [7]. However, the distribution of serotypes can vary with age, geography and time [8], which increases the challenges for vaccine development.

Pneumococcal conjugate vaccines prevent invasive pneumococcal disease, including pneumonia. Since the introduction of polysaccharide-based pneumococcal conjugated vaccines, a significant decline in invasive pneumococcal disease caused by vaccine-targeted serotypes has been observed in young children, and also in non-vaccinated children of the same age group via herd immunity [9]. However, reports indicate some replacement of vaccine types by non-vaccine types, both among carriage and invasive isolates [10]. An increase in the frequency of disease caused by previously uncommon non-vaccine types could necessitate changes in vaccine composition, emphasizing the need for continued surveillance [10]. Changes in bacterial seroepidemiology need to be constantly monitored to evaluate pneumococcal population biology and transmission, as well as the efficacy and appropriateness of newer vaccines.

Historically, serotype identification has been performed using serological methods such as the Quellung test [11], which remains the gold standard. However, this method is labor-intensive and requires a certain level of experience to be performed correctly, and is therefore mainly used in specialized reference or research laboratories [12]. Other current antibody-based pneumococcal serotyping techniques, such as the latex agglutination test or lateral flow assays [13], are not satisfactory due to their lack of sensitivity and specificity and the fact they are usually labor-intensive [14]. However, first and foremost, all of these methods require prior isolation and culture of the bacterium and are therefore slow, time-consuming, subject to varied interpretation, and usually cannot detect multiple serotypes simultaneously. Molecular serotyping techniques using real time PCR have been described but target a limited number of serotypes and provide insufficient quantitative information on bacterial load [15–17].

The relationship between the bacterial count in the blood samples of patients and the severity of pneumococcal disease has already been described [18–21]. Moreover, the bacterial load in the upper respiratory tract of healthy carriers is potentially related to the risk of developing an invasive infection [22]. In order to determine the complex relationship between capsule composition, serotype-specific load, the risk of infection for healthy carriers and disease severity, we developed a real-time multiplex PCR method to accurately serotype and quantify the bacterial concentration of 40 globally-prevalent *S*. *pneumoniae* serotypes. The assay targets genomic regions specific to distinct *S*. *pneumoniae* serotypes, which have already been described in serotyping studies, using conventional multiplex PCR [23]. Microbiological diagnosis of pneumococcal disease in young children is a challenge, as only low volumes of clinical samples, especially blood, are usually available for laboratory diagnosis. The multiplex PCR assay developed in this study was optimized to obtain good specificity and sensitivity based on analysis of as little as 200 μl of clinical sample. The 40 most frequently represented serotypes or serogroups (1, 2, 3, 4, 5, Sg6, 6C, 7C/(7B/40), 7F/(7A), 8, 9N/L, 9V/(9V), 10A, 10F/(10C),11A/(11D), 12F/(12A/12B/44/46), 13, 14, 15A/(15F), 15B/C, 16F, 17F, Sg18, 19A, 19F, 20, 21, 22F/(22A), 23A, 23B, 23F, Sg24, 31, 33F/(33A/37), 34, 35A/(35C/42), 35B, 35F/(47F), 38/(Sg25), 39) were tested using 11 multiplex real-time PCR. The use of external quantified calibrators enables quantification of the bacterial concentration for each specific serotype. We first validated the specificity and sensitivity of the assay using well-characterized and quantified *S*. *pneumoniae* strains representing the 40 serotypes. The assay was then evaluated using clinical nasopharyngeal (NP) and whole blood (WB) samples from five different cohorts of patients hospitalized for pneumonia in Cambodia, Mali, France, Brazil or South Africa.

The multiplex real-time PCR typing assay described in this study enables rapid, direct *S*. *pneumoniae* serotype identification and quantification using clinical samples. Moreover, this study indicates the assay has the ability to predict the invasiveness of a serotype based on the bacterial concentrations of the non-invasive serotypes carried in the nasopharynx.

## Materials and Methods

### Bacterial strains

One hundred and seventeen pneumococcal strains including the 40 targeted serotypes, previously isolated from clinical specimens and characterized using the Neufeld Quellung method, were provided by the National Reference Center for Streptococci, University Hospital RWTH Aachen, Germany, and used to develop the test. The strains were: [1, 3, 4, 6A, 6B, 9V, 10A, 11A, 12F, 13, 15A, 15B, 15C, 16F, 19A, 19F, 22F, 33F, 34, 35B] (n = 3); [2, 9L, 9N, 18F, 21, 23A, 23B, 24F, 35A] (n = 2); [5, 6C, 7A, 7B, 7C, 7F, 8, 9A, 10C, 10F, 11D, 12A, 12B, 14, 15F, 17F, 18A, 18B, 18C, 20, 22A, 23F, 24A, 24B, 25A, 25F, 31, 33A, 33D, 35C, 35F, 37, 38, 39, 40, 42, 44, 46, 47F] (n = 1). In this assay, the isolates 6A, 6B and 6C, 9L and 9N, 15B and 15C, 18 and 24 are considered as serogroups Sg6, 9NL 15BC, Sg18 and 24, respectively.

Twenty nine isolated strains representatives of 29 different *Streptococcus* species provided by bioMérieux, France, were used: *S*. *agalactiae*, *S*. *anginosus*, *S*. *australis*, *S*. *bovis*, S. canis, *S*. *constellatus ssp constellatus*, *S*. *constellatus ssp pharynges*, *S*. *cristatus*, *S*. *downei*, *S*. *ferus*, *S*. *gordonii*, *S*. *infantarius ssp coli*, *S*. *infantarius ssp infantarius*, *S*. *infantis*, *S*. *iniae*, *S*. *intermedius*, *S*. *mitis*, *S*. *oralis*, *S*. *mutans*, *S*. *oligofermentans*, *S*. *parasanguinis*, *S*. *peroris*, *S*. *pseudopneumoniae*, *S*. *pyogenes*, *S*. *ratti*, *S*. *salivarius ssp salivarius*, *S*. *sanguinis*, *S*. *sinensis*, *S*. *sobrinus*.

### Media and culture conditions

All pneumococcal strains were retrieved from -80°C storage and subcultured in 8 ml of Todd-Hewitt broth with 0.5% yeast extract at 37°C in 5% CO_2_ at an initial OD_600nm_ between 0.04 and 0.06. The OD_600nm_ was measured every hour and cultures were harvested at an OD_600nm_ between 0.4 and 0.6.

### Bacterial concentration

The bacterial concentration of the standard cultures was estimated by spreading ten-fold serial dilutions on Columbia agar containing 5% sheep blood (bioMérieux, France) and colony counting after 20h of growth at 37°C and 5% CO_2_.

### Cohorts

Hospitalized patients from five different geographical areas were enrolled by pediatricians and/or clinicians. The South African cohort was composed of 400 NP samples and 322 WB samples from HIV+ or HIV- adults hospitalized with symptoms of pneumonia between 2006 and 2007 [22,24]. All WB samples had previously been tested for serotype identification on-site using the Quellung serotyping test [11]. The samples from the second cohort were provided by Bonsucesso Geral Hospital and Gaffre Guinle Hospital, which are both located in the city of Rio de Janeiro, Brazil. This cohort included 123 NP samples from children admitted with a diagnosis of lower respiratory tract infection (LRTI), including bronchiolitis or pneumonia, in the period from 2006 to 2009. The cohort from Mali included 117 children under 5 years-of-age hospitalized with symptoms of pneumonia at the Gabriel Touré Hospital, Bamako, Mali between 2011 to 2012; a NP sample was collected from each patient and WB samples were available for 110 of these patients [25,26]. The cohort from Cambodia was composed of 211 children under 5 years-of-age, recruited from patients hospitalized with symptoms of pneumonia at the National Pediatric Hospital, Phnom Penh, Cambodia between 2010 to 2013 [25]. The final cohort comprised 63 children hospitalized for community acquired pneumonia at the emergency unit and pediatric intensive care unit of Hospices Civils de Lyon, France, between 2007 and 2009; 63 NP aspirates, and 5 WB samples that tested positive for *S*. *pneumoniae* by blood culture were available for this cohort [27].

At the Emerging Pathogens Laboratory, Fondation Mérieux, in Lyon, all NP samples from the five cohorts were tested using the Fast Track Diagnostic (FTD) respiratory 21 plus test (Fast-track Diagnostics, Luxembourg, Luxembourg), which detects 19 respiratory viruses and five bacteria including *S*. *pneumoniae*. The direct blood samples (not cultured) were tested using a homemade real-time triplex PCR assay, which enables the detection of three bacteria including *S*. *pneumoniae* [22,24]. A total of 664 samples found positive for *S*. *pneumoniae* including 562 NP samples (South Africa, *n =* 227; Mali, *n =* 86; Cambodia, *n =* 149; Brazil, *n =* 55; France, *n =* 45) and 102 WB samples (South Africa, *n =* 81; Mali, *n =* 16; France, *n =* 5) were used for the study. NP and WB samples from the same patient (*n =* 88) were available for 67 South African patients, 16 Malian patients and 5 French patients.

### Ethics and confidentiality

Medical written informed consent was obtained from all adult patients and from parents or tutors for children. The study protocol, informed consent statement, clinical research form, any amendments and all other study documents were submitted to and approved by the ethics committee of each site: Brazil (Conselho Nacional de Saúde, Comissão Nacional de Ética em Pesquisa, Ministerio da Saúde), Cambodia (National Ethics Committee for Health Research, Ministry of Health), Mali (Comité national d'éthique pour la santé et les sciences de la vie), South Africa (ethics committees of the University of Witwatersrand and Emory University), France (Comité Consultatif National d’Ethique, approval reference number DGS2007-0022MS-01).

### Nucleic acid extraction

Nucleic acids were extracted from 200 μl of the NP samples using an easyMAG automate (bioMérieux, France) and eluted in 110 μl of elution buffer, according the manufacturer’s recommendations. DNA was extracted from 200 μl of the direct blood samples (not cultured) using the QIAamp^®^ DNA Blood Mini Kit (Qiagen, Netherlands), following the manufacturer’s recommendations, and eluted in 100 μl of elution buffer.

### Primers and probes design

Primers and probes were newly designed based on the regions previously described by Pai and col (2006) to enable the detection of the 40 most prevalent *S*. *pneumoniae* serotypes worldwide [23]. Nucleotide sequences were retrieved from the GenBank database. Oligonucleotide primer and probe sequences were designed in divergent loci of the capsular gene cassette to target serotype-specific regions. To avoid theoretical false positive rates, primers and probes for each set of serotype were aligned with all available serotype sequences from GenBank database. Only the most specific primer and probe serotype sets were selected. The multiplex PCR assay described in this study also includes an internal positive control targeting the *lytA* gene, a gene conserved among pneumococci. The primers and probes targeting the *lytA* gene are specific for *S*. *pneumoniae* and are not complementary to other related bacteria. The primer pairs and probes specific to each of the 40 serotypes were grouped in 11 multiplex reactions on the basis of the following criteria: internal primer binding properties for hairpin and primer-dimer potential, length of the desired amplicon, G-C content, and melting temperatures (T_m_) of the probes and primers. Four different fluorophores were used to label the probes at the 5’ end: carboxy-fluorescein (FAM), hexachloro-6-carboxy-fluorescein (HEX), Texas red (sulforhodamine 101 acid chloride) or Cyanine 5, with two different black hole quenchers at the 3’ end: BHQ1 and BHQ2. Locked nucleic acid (LNA) chemistry was used for the probe design [28]. Beacon Designer^™^ software (PREMIER Biosoft International, Palo Alto, USA) was used to design the optimal multiplex PCR combinations using a maximum of four probes and primer pairs per multiplex reaction. The primers and probes sequences, their concentration and the composition of the pools used in the molecular quantitative typing assay are detailed in Table 1.

**Table 1:** Primers/Probes Sequences and Concentration Used in Each Pool for the *S*. *pneumoniae* Serotype Identification and Quantification Assay.

Multi-plex PCR	Oligo-nucleotide	Sequence (5’-3’)*	Probe dye (5’)	Probe quencher (3’)	Final concentration (nM)	Gene	GenBank accession number	Nucleotide position
1	19A-F^a^	ACTACTGTTCAAGAGGATTATACAC	-	-	80	*mnaA*	CR931675	12 639
1	19A-R^b^	CATTCAATACTCGTTTAACTGCTC	-	-	80	*mnaA*	CR931675	12 757
1	19A-P^c^	TGTcTCaTCGGTtCGCC	Cy5	BHQ2	80	*mnaA*	CR931675	12 732
1	22F-F	ATTTACCCATCATCACAACTATTTG	-	-	80	*wcwV*	CR931682	11 583
1	22F-R	TACCGTTTATACCATCTTGAATCAG	-	-	80	*wcwV*	CR931682	11 706
1	22F-P	CTCtCCtCCaGCaCTTGC	HEX	BHQ2	80	*wcwV*	CR931682	11 685
1	3-F	GAAAACAAGGAAAGATGGAACTTC	-	-	80	*galU*	CR931634	8 738
1	3-R	ATTACAACAAAGGGATCGTCAC	-	-	80	*galU*	CR931634	8 864
1	3-P	AGAaCAgCGtCAcCAAGA	FAM	BHQ1	80	*galU*	CR931634	8 826
1	6AB-F	AGATGGTTCCTTCAGTTGATATTG	-	-	80	*wciP*	CR931639	8 720
1	6AB-R	ATTATGTCCATGTCTTCGATACAAG	-	-	80	*wciP*	CR931639	8 844
1	Sg6-P	CTCaGGgCAgAAcAACAC	TR	BHQ2	80	*wciP*	CR931639	8 818
2	14-F	CGTCTTTTTGTATGGTGCTATG	-	-	160	*wzy*	CR931662	7 261
2	14-R	GCCAATTAAATGCTTTACTCAAATC	-	-	160	*wzy*	CR931662	7 356
2	14-P	ACAcTTgAAcAGcCAATCC	Cy5	BHQ2	160	*wzy*	CR931662	7 329
2	4-F	GGGGAAGTATTTTCAGAGTCG	-	-	160	*wzy*	CR931635	10 479
2	4-R	AATCACCAACTAACCATCCAATAG	-	-	160	*wzy*	CR931635	10 603
2	4-P	TGAcCAgCCtAAcAGTAGC	HEX	BHQ2	160	*wzy*	CR931635	10 504
2	12F-F	TGATATGCGTGAATCTCCAAC	-	-	160	*mnaB*	CR931660	15 309
2	12F-R	ACTGATTCGCCACAACATC	-	-	160	*mnaB*	CR931660	15 406
2	12F-P	AGAaTCtATgAGcCGCCA	FAM	BHQ1	160	*mnaB*	CR931660	15 342
2	9V-F	TCCTCAGTCAATTTTAACAAGAAAC	-	-	160	*wzx*	CR931648	12 130
2	9V-R	AGAGAATATACCCCGAAATCATG	-	-	160	*wzx*	CR931648	12 233
2	9V-P	CCAgCAcAAcACcAATAAC	TR	BHQ2	160	*wzx*	CR931648	12 209
3	23F-F	TAGTGACAGCAACGACAATAG	-	-	80	*wzy*	CR931685	9 045
3	23F-R	AAAACAAATGAAACCTATCTGATTC	-	-	80	*wzy*	CR931685	9 170
3	23F-P	TCAcAAcACcTAaCACTCG	Cy5	BHQ2	80	*wzy*	CR931685	9 134
3	11A-F	CATTGTGTATGCTACCATTCTTC	-	-	80	*wzy*	CR931653	11 987
3	11A-R	GTGCTAACTGTAAAACTTGATTATG	-	-	80	*wzy*	CR931653	12 058
3	11A-P	TCTcCCaATtTCtGCCACG	HEX	BHQ2	80	*wzy*	CR931653	12 096
3	33F-F	AGATTAGATGGTTTGCTGGAC	-	-	80	*wzy*	CR931702	10 968
3	33F-R	CAGAATAACTGATACCACAAGTAAC	-	-	80	*wzy*	CR931702	11 078
3	33F-P	AGTcGCcCAtTTcCCT	FAM	BHQ1	80	*wzy*	CR931702	11 060
3	7F-F	TTGATGAAGGCTTTGGTTTG	-	-	80	*wcwH*	CR931643	14 098
3	7F-R	CCATCAATTGCATATTCAAATACAC	-	-	80	*wcwH*	CR931643	14 173
3	7F-P	ACTaACgCAcAGcCACAA	TR	BHQ2	80	*wcwH*	CR931643	14 138
4	16F-F	TTCGCAAGGAGAGATTACTG	-	-	80	*wzy*	CR931668	11 610
4	16F-R	TTGGGAAGGATATCCTATTTTAATC	-	-	80	*wzy*	CR931668	11 716
4	16F-P	TTTaACaCCcACgCCTGAA	Cy5	BHQ2	80	*wzy*	CR931668	11 685
4	35B-F	ATTGCAAATATTCGACTACCTAG	-	-	160	*wcrH*	CR931705	10 862
4	35B-R	ATATGAACTTTTTCCTTTTGCTAG	-	-	160	*wcrH*	CR931705	10 938
4	35B-P	CCGcTAtAAcCAcTCCATC	HEX	BHQ2	160	*wcrH*	CR931705	10 914
4	Sg18-F	TGGCTAGAACAGATTTATGGAAG	-	-	80	*wzy*	CR931673	12 938
4	Sg18-R	AAAGTCATCCAATTATTATCCATCC	-	-	80	*wzy*	CR931673	13 028
4	Sg18-P	CAAaCCcTAtCCcTCTCCC	FAM	BHQ1	80	*wzy*	CR931673	13 000
4	19F-F	CTTTACAGGAAGGAAATATACAACC	-	-	80	*wzy*	CR931678	11 216
4	19F-R	CGTCAATAACACGAATGAGAAC	-	-	80	*wzy*	CR931678	11 339
4	19F-P	TCGcACtGTcAAtTCACC	TR	BHQ2	80	*wzy*	CR931678	11 309
5	15B/C-F	TTTGCTACAGGTTTTAGTATTGAG	-	-	80	*wzy*	CR931665	7 701
5	15B/C-R	AAAGCAATATAAGAGGTATAGTTGG	-	-	80	*wzy*	CR931665	7 825
5	15B/C-P	CGCtACaATcATcCGCT	Cy5	BHQ2	80	*wzy*	CR931665	7 802
5	31-F	TTAAGAATCTTGTTTCGAATGAGAC	-	-	80	*wzy*	CR931695	10 003
5	31-R	TGTTAAAAGAGTACTCTGATACCAG	-	-	80	*wzy*	CR931695	10 103
5	31-P	AACaCAcTCgCCgATAAA	HEX	BHQ2	80	*wzy*	CR931695	10 073
5	38-F	GAAGCCTACCTACAATAACAGTG	-	-	80	*wzy*	CR931710	14 221
5	38-R	CAGCCATTATACTCCAACCTAAG	-	-	80	*wzy*	CR931710	14 336
5	38-P	TGCtCCaAGtTTcCTCAG	FAM	BHQ1	80	*wzy*	CR931710	14 299
5	8-F	AGGAGCCTATGATTTAATAGATGC	-	-	80	*wciS*	CR931644	8 065
5	8-R	TTGATATCTTTTGACGTATGTCTTC	-	-	80	*wciS*	CR931644	8 153
5	8-P	TTCtCCaTCtCCaGCCATT	TR	BHQ2	80	*wciS*	CR931644	8 131
6	10A-F	GGGAGTCTACAATTACTTCGC	-	-	80	*wcrG*	CR931649	12 256
6	10A-R	GCATAACGAAATCCTAACATCTC	-	-	80	*wcrG*	CR931649	12 360
6	10A-P	TCCaGTgCAaTAcATTCCA	Cy5	BHQ2	80	*wcrG*	CR931649	12 282
6	35F-F	ATTAGGTGGTCGTATATACTTGATG	-	-	80	*wzy*	CR931707	7 689
6	35F-R	ACATACAGATAGGTCTGATAGTTTC	-	-	80	*wzy*	CR931707	7 799
6	35F-P	TCAgACcATtTCcATTCCA	HEX	BHQ2	80	*wzy*	CR931707	7 765
6	1-F	TACGGGGTATTCTAATCAAACTTG	-	-	80	*wzy*	CR931632	10 651
6	1-R	AGCCTGTTAGCATAAAAATCATTAC	-	-	80	*wzy*	CR931632	10 769
6	1-P	TCAcATaTCcCTcTCCCAC	FAM	BHQ1	80	*wzy*	CR931632	10 730
6	34-F	CTTATTGTTGTAGTGGCAGTTG	-	-	80	*wzy*	CR931703	8 034
6	34-R	CTTGAATAGTTCTCGATTAAGAGC	-	-	80	*wzy*	CR931703	8 127
6	34-P	CCAtCTtGAcCTaCTCCA	TR	BHQ2	80	*wzy*	CR931703	8 099
7	15A-F	CGTTATTTAGTGAATTGCTATACTC	-	-	160	*wzy*	CR931663	7 160
7	15A-R	TCCCTGCAGAATAAGAATCTAC	-	-	160	*wzy*	CR931663	7 255
7	15A-P	TACtGCtGCtGCcAACA	Cy5	BHQ2	160	*wzy*	CR931663	7 229
7	17F-F	TGATTCGTGATGATAATTCCAATG	-	-	160	*wciP*	CR931670	10 481
7	17F-R	TTTGTCTAACATCGTTTAATAACCC	-	-	160	*wciP*	CR931670	10 606
7	17F-P	AGGcTCcATgATaTTCCGA	HEX	BHQ2	160	*wciP*	CR931670	10 573
7	20-F	GAGGATGAACTTATTTCTAAAGGAG	-	-	160	*wciL*	CR931679	9 545
7	20-R	TCTTTGAAGAATCTATACATTTCCC	-	-	160	*wciL*	CR931679	9 621
7	20-P	TTAcTTcTCgCTgTCAGGT	FAM	BHQ1	160	*wciL*	CR931679	9 582
7	7C-F	TTTCAACAACGGAAGGTTTTG	-	-	80	*wcwL*	CR931642	9 772
7	7C-R	CTTCTGTTATTAATTCTGGACTAGC	-	-	80	*wcwL*	CR931642	9 852
7	7C-P	CACaAAgACcGTtCGCT	TR	BHQ2	80	*wcwL*	CR931642	9 815
8	lytA-F	GCGGAAAGACCCAGAATTAG	-	-	160	*lytA*	M13812	712
8	lytA-R	GAATGGCTTTCAATCAGTTCAAC	-	-	160	*lytA*	M13812	839
8	lytA-P	TCTcAGcATtCCaACCGC	FAM	BHQ1	160	*lytA*	M13812	810
8	5-F	AGTAGCGGATTGCTGATTTAC	-	-	160	*wzy*	CR931637	6 585
8	5-R	ACGTCTCCTGAAACCATATATAAAC	-	-	160	*wzy*	CR931637	6 663
8	5-P	CTAaCAgAAcTGcTCGTGA	TR	BHQ2	160	*wzy*	CR931637	6 624
9	23B-F	AATCTGCTTTGGTTGGAATG	-	-	80	*wzx*	CR931684	13 524
9	23B-R	AACTAGCTATCATAGTTGAGATTG	-	-	80	*wzx*	CR931684	13 602
9	23B-P	ATCcCCtGTaGTcCCAT	Cy5	BHQ2	80	*wzx*	CR931684	13 583
9	35A-F	ATCATAAGGTAGTCAATAAGATGC	-	-	80	*wzx*	CR931704	15 139
9	35A-R	TGAACAAAATCATCATTCACAATC	-	-	80	*wzx*	CR931704	15 221
9	35A-P	ATCcCTgCCaTAaTCGG	HEX	BHQ2	80	*wzx*	CR931704	15 201
9	6C-F	TTTTGTTATTGTCGGCGATG	-	-	80	*wciNβ*	EU714777	2 094
9	6C-R	ATTGAACTGAGCTAAATAATCCTC	-	-	80	*wciNβ*	EU714777	2 200
9	6C-P	TTAtCCaCCcACcCTGT	FAM	BHQ1	80	*wciNβ*	EU714777	2 179
9	9N/L-F	AATTGTACCGCAAGCTATTC	-	-	80	*wzx*	CR931647	12 121
9	9N/L-R	TTAGAGAATAGACACCAAAATGTG	-	-	80	*wzx*	CR931647	12 231
9	9N/L-P	AATaACgCCcACcAAG	TR	BHQ2	80	*wzx*	CR931647	12 205
10	10F-F	ATCCTTCTGATTTTGGTAACTTCC	-	-	80	*wzx*	CR931652	12 587
10	10F-R	TGAGGAAGGACATCTCATGC	-	-	80	*wzx*	CR931652	12 682
10	10F-P	CCGcTTtGTgTTcACCT	Cy5	BHQ2	80	*wzx*	CR931652	12 655
10	2-F	CTGCCTATATTTTGATGTGTTTGC	-	-	80	*wzy*	CR931633	10 543
10	2-R	TTCTAAGAGTTCCAATACGTTGAC	-	-	80	*wzy*	CR931633	10 638
10	2-P	AACcATcAGcCAgTCCA	HEX	BHQ2	80	*wzy*	CR931633	10 615
10	23A-F	CTATTCTAGCAAGTGACGAAGATG	-	-	80	*wzy*	CR931683	7 738
10	23A-R	GCAGAACTTGTAGTGTGACAG	-	-	80	*wzy*	CR931683	7 846
10	23A-P	ATCcGCtCCaAAtCCCA	FAM	BHQ1	80	*wzy*	CR931683	7 819
10	Sg24-F	TGTGGTTTTCAGGACTTATTGC	-	-	80	*wzy*	CR931688	11 664
10	Sg24-R	TTGACTTTATCATAGGTCGGAAAG	-	-	80	*wzy*	CR931688	11 766
10	Sg24-P	CAAgGAaAAgGGcTCCC	TR	BHQ2	80	*wzy*	CR931688	11 690
11	13-F	AGGTGTAATCTCTATCTTCC	-	-	320	*wzx*	CR931661	14 260
11	13-R	AAATGATCTCTACCAATAAATTC	-	-	320	*wzx*	CR931661	14 375
11	13-P	AGGcAAcCAcATaCTTA	Cy5	BHQ2	320	*wzx*	CR931661	14 352
11	21-F	AAGTGATTATAAGTCTGTGAAG	-	-	160	*wzx*	CR931680	13 937
11	21-R	AAAAGAATTGAACAAAGTCG	-	-	160	*wzx*	CR931680	14 036
11	21-P	ATCaACaTCcCAgCAA	HEX	BHQ2	160	*wzx*	CR931680	14 011
11	39-F	CAGTCTTATTACTCCCAATAG	-	-	160	*wzy*	CR931711	11 239
11	39-R	AAATAAATAAGCTGCTTTATAATG	-	-	160	*wzy*	CR931711	11 364
11	39-P	AGGcTCcATcATcAGTA	FAM	BHQ1	160	*wzy*	CR931711	11 345

* Nucleotides indicated in lower cases correspond to LNA-modified bases

^a^ F, forward primer;

^b^ R, reverse primer;

^c^ P, probe

### Multiplex PCR amplifications

The 40 *S*. *pneumoniae* serotype real-time PCR typing assay (40-PCR) was performed in eleven multiplex tubes among which one includes the *lytA* positive control. Real-time multiplex PCR (Taqman) reactions were amplified on a CFX96 thermocycler (BioRad, Hercules, CA, USA). The reaction mixtures contained 5 μl of template DNA, 1X BioRad iQ Powermix (BioRad) and 80–320 nM of each primer and probe (depending on the primer-probe set), made up to a final volume of 50 μl. Reaction conditions were as follows: 95°C for 5 min followed by 40 cycles of 95°C for 15 sec and 60°C for 60 sec. Curves analyses and *S*. *pneumoniae* serotype identification were performed using CFX Manager Software version 3.1 (Bio-Rad). The results were expressed as the cycle threshold (C_T_).

### *S*. *pneumoniae* serotype-specific load quantification

DNA purified from standard cultures of the corresponding serotypes was serially diluted and used to perform *S*. *pneumoniae* serotype-specific real-time PCR in order to establish the relationship between the cycle threshold (C_T_) value and bacterial concentration for each serotype. The bacterial concentration of the standard cultures was calculated by colony counting on blood agar plates and expressed in colony forming units per milliliter (CFU/ml). The bacterial load of the clinical samples was estimated by comparing their C_T_ values to the standard curves obtained using the serially-diluted standard cultures. When a serotype was identified, the sample was tested again with the corresponding serotype-specific multiplex PCR. Ten-fold serial dilutions of the quantified serotype strain from 10^7^ to 10^2^ CFU/ml were extracted and amplified in parallel in the same PCR run and used as a calibrator. Serotype quantification for each sample was expressed as CFU/ml.

### Statistical analysis

Categorial variables were described as number and percentage, continuous variables were described as mean (± standard deviation) or median (interquartile range [IQR]). The Student's *t-*test was used to compare continuous variables after checking that the application conditions concerning normality and variances distributions are met. The sensitivity of the quantitative multiplex real-time PCR assay compared with colony counting was tested for each serotype using linear regressions and calculation of coefficient of determination (R^2^). The results were considered statistically significant at *P*-values of less than 0.05 (*P* < 0.05), all the tests were 2-tailed, Stata 13.0 (Stata Corp) was used for analysis.

## Results

### Set up and optimization of the quantitative pneumococcal serotype-specific molecular assay

The 44 *S*. *pneumoniae* reference strains were cultivated in liquid growth medium and harvested for DNA extraction in the late exponential growth phase in order to limit the number of non-viable bacteria. In parallel, ten-fold serial dilutions were plated onto Columbia blood agar plates and colony numbers were associated with the qPCR results to determine the correlation between the bacterial concentration and PCR cycle threshold (C_T_) value for each serotype.

The optimal PCR conditions were determined by testing ten-fold serial dilutions of the DNA templates with a range of oligonucleotide concentrations (80 to 800 nM). For most primer-probe sets, 80 nM of each oligonucleotide appeared to be the optimal concentration in terms of detection limits. At concentrations higher than 320 nM, the sensitivity of the test was affected as at low template concentrations, fluorescence intensity was weak and the amplification curves were difficult to discriminate from non-specific signals. Furthermore, the use of equal concentrations of primers and probe provided stronger fluorescence intensity without affecting the sensitivity of the test.

In the current methodology, a total of 23 primer-probe sets were demonstrated to be completely specific for the targeted serotypes in the tested strains from the National Reference Center for Streptococci, Aachen, Germany, which were previously validated by the Quellung serotyping test. The serotyping results perfectly matched with that of the Quellung serotyping test. However, it was not possible to discriminate serotypes among some serogroups: serogroup 6 (6A, 6B and 6C), 7 (7A and 7F, 7B and 7C), 9 (9A and 9V, 9N and 9L), 10 (10C and 10F), 11 (11A and 11D), 12 (12A, 12B and 12F), 15 (15A and 15F, 15B and 15C), 18 (18A, 18B, 18C and 18F) 22 (22A and 22F), 24 (24A, 24B and 24F), 33 (33A and 33F) and 35 (35A and 35C). Cross reactions between different serogroups and/or serotypes were observed: serotype 38/serogroup 25, serogroup 12/serotypes 44 and 46, serotypes 33A/F/serotype 37, serotypes 35A/C/serotype 42, serotype 35F/serotype 47F and serotypes 7B/C/serotype 40. The *lytA* positive control gene was also correctly detected for all pneumococcal serotypes. Based on primers and probes sequences alignments, the Sg6 primers and probe set detects also theoretically the serotypes 6D and 6E. The serotype 6D can however be detected by the 6C primers. However, as tested, the 6C primers and probe set included in the multiplex PCR pool 9 allows the specific discrimination of the serotype 6C from the Sg6. The cross reactions observed are clarified in the Table 2. Moreover, no cross-reactivity was observed with any other Gram-positive species tested as detailed in the Material and Method chapter. None of the 29 *S*. *pneumoniae*-related bacteria were detected by the *lytA* or *S*. *pneumoniae* serotype-specific amplifications (data not shown).

**Table 2:** Cross Reactions Observed for Each Multi-plex PCR.

Multi-plex PCR	Serotype detected	Cross Reaction observed
1	19A	-
1	22F	22A
1	3	-
1	6AB	6CDE
2	14	-
2	4	-
2	12F	12AB/44/46
2	9V	9A
3	23F	-
3	11A	11D
3	33F	33A/37
3	7F	7A
4	16F	-
4	35B	-
4	Sg18	-
4	19F	-
5	15B/C	-
5	31	-
5	38	Sg25
5	8	-
6	10A	-
6	35F	47F
6	1	-
6	34	-
7	15A	15F
7	17F	-
7	20	-
7	7C	7B/40
8	LytA	-
8	5	-
9	23B	-
9	35A	35C/42
9	6C	6D
9	9N/L	-
10	10F	10C
10	2	-
10	23A	-
10	Sg24	-
11	13	-
11	21	-
11	39	-

The sensitivity of the quantitative multiplex real-time PCR assay was evaluated by analyzing ten-fold serial dilutions of purified nucleic acids from each pneumococcal strain, and the corresponding bacterial concentrations were determined on the basis of colony counting on blood agar plates. The sensitivity for each serotype is detailed in the Table 3. The lower detection limit of the quantitative multiplex real-time PCR assay for each serotype was defined as the lowest concentration of the serially-diluted standards detected by PCR with a C_T_ value ≤ 35. The C_T_ values > 35 were considered as negative results. Variable analytical sensitivity was observed for the different serotypes. Depending on the serotype, the analytical sensitivity ranged from 1 to 455 CFU/reaction, for serotypes [9V, 18C, 10A, 5] and 35B respectively. For 40 of the 44 strains (91%) used in this study, the assay was highly sensitive as less than 100 CFU/reaction could be detected. For 4 isolates (9%) the lowest bacterial concentration detected ranged between 100 and 455 CFU/reaction.

**Table 3:** Serotype-Dependent Detection Limits and Linear Equation Values.

Multi-plex PCR	Serotype	Lowest detection limit (CFU*)	Linear Equation	R^2^
1	3	5	-0.2760x + 9.9654	0.9884
1	22F	136	-0.2935x + 11.841	0.9834
1	6A	12	-0.2966x + 10.446	0.9942
1	6B	18	-0.3083x + 11.240	0.9991
1	19A	6	-0.2914x + 10.467	0.9908
2	12F	10	-0.3064x + 10.970	0.9957
2	4	8	-0.3050x + 10.523	0.9985
2	9V	1	-0.3306x + 11.154	0.9845
2	14	11	-0.3046x + 10.908	0.9934
3	33F	10	-0.3229x + 12.364	0.9791
3	11A	64	-0.2775x + 9.8794	0.9826
3	7F	8	-0.3048x + 10.652	0.9972
3	23F	20	-0.2891x + 11.258	0.9936
4	18C	1	-0.2646x +10.475	0.9980
4	35B	455	-0.2982x + 10.322	0.9931
4	19F	3	-0.2762x + 9.7279	0.9989
4	16F	5	-0.2969x + 10.309	0.9955
5	38	5	-0.2871x + 10.324	0.9909
5	31	7	-0.3047x + 10.657	0.9986
5	8	7	-0.3334x + 12.649	0.9803
5	15B	9	-0.2868x + 10.502	0.9982
5	15C	84	-0.2885x + 12.026	0.9987
6	1	16	-0.2881x + 10.89	0.9965
6	35F	10	-0.2904x + 10.288	0.9963
6	34	10	-0.2824x + 10.342	0.9956
6	10A	1	-0.3266x + 11.581	0.9917
7	20	11	-0.3045x + 10.600	0.9962
7	17F	10	-0.3049x + 10.660	0.9912
7	7C	15	-0.3085x + 10.887	0.9916
7	15A	3	-0.3097x + 10.426	0.9914
8	5	1	-0.2763x + 9.7484	0.9949
9	6C	10	-0.3277x + 13.258	0.9959
9	35A	158	-0.3052x + 12.141	0.9931
9	9N	352	-0.2952x + 13.369	0.9977
9	9L	50	-0.2885x + 12.122	0.9991
9	23B	43	-0.2515x +10.763	0.9892
10	23A	61	-0.3479x +12.659	0.9947
10	2	63	-0.2661x + 11.295	0.9972
10	24F	2	-0.3171x + 12.581	0.9946
10	10F	83	-0.3072x + 12.638	0.9961
11	39	2	-0.2977x + 11.967	0.9970
11	21	21	-0.3264x + 12.790	0.9986
11	13	20	-0.3185x + 12.594	0.9986

* CFU values expressed as numbers per PCR

### Clinical evaluation of the typing assay in five cohorts of patients with pneumonia

The 40-PCR assay was evaluated on five different cohorts of patients diagnosed with CAP from four different continents and positive for *S*. *pneumoniae*.

Overall, 89% of the NP samples (502/562) were positive for at least one *S*. *pneumoniae* serotype, whereas one or more *S*. *pneumoniae* serotypes were identified in 64% (65/102) of the WB samples. A single positive detection was obtained in 72.9% (366/502) of the NP samples, two in 21.1% (106/502), three in 3.4% (17/502) and more than three in 2.6% (13/502). One single serotype was identified in 43.8% (220/502) of the NP samples. One positive detection or two were found in 96.9% (62/65) and 3.1% (2/65) of the WB samples, respectively. One single serotype was identified in 75.4% (49/65) of the WB samples.

Among the 88 WB samples for which the corresponding NP sample was available, 57 samples were positive for at least one *S*. *pneumoniae* serotype in the 40-PCR assay. In 82.4% (47/57) of these cases, the serotype found in the WB sample was also among the serotypes found in the corresponding NP sample. In cases where several serotypes were present in the NP sample, the serotype with the highest bacterial load was the serotype found in the corresponding WB sample.

**Fig 1:**
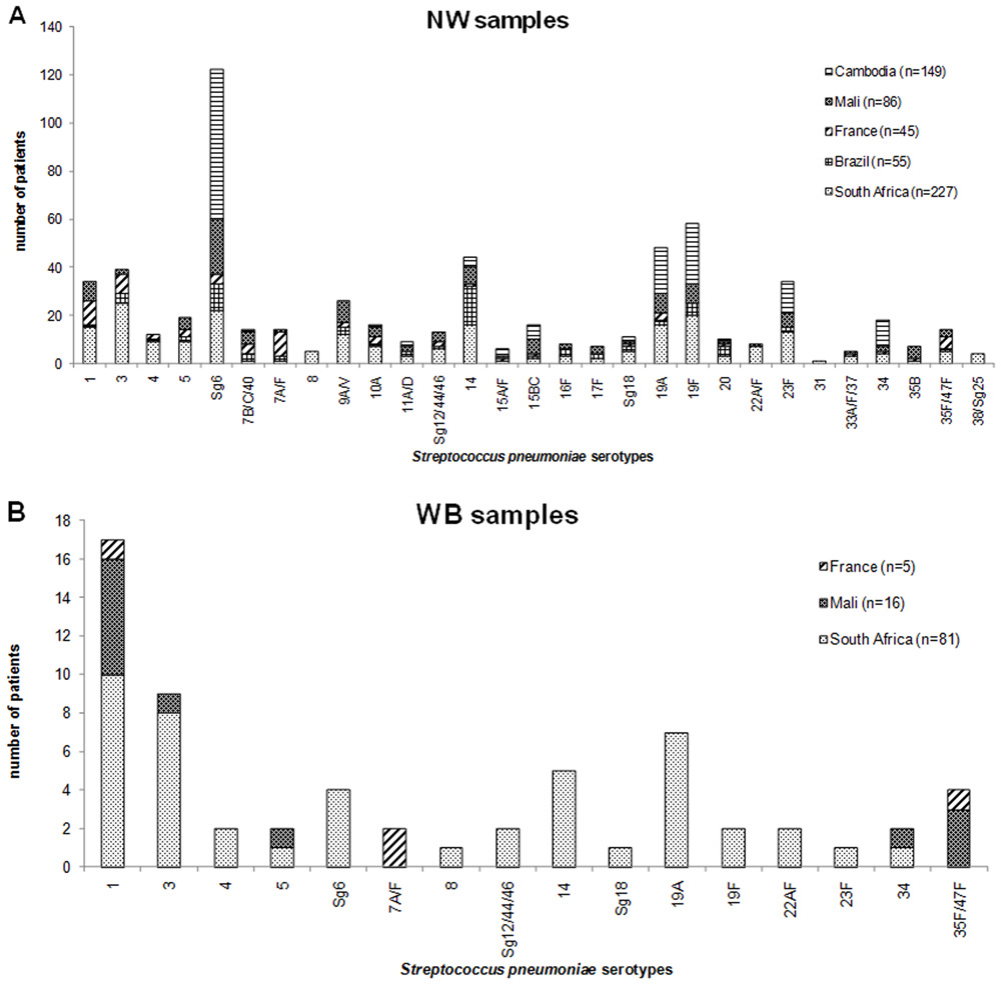
*S*. *pneumoniae* serotypes distribution determined by the 40 *S*. *pneumoniae* serotype real-time PCR typing assay (40-PCR). (A) in NP samples (n = 562) of patients from Cambodia (n = 149), France (n = 45), South Africa (n = 227), Mali (n = 86) and Brazil (n = 55), (B) in WB samples (n = 102) of patients from France (n = 5), Mali (n = 16) and South Africa (n = 81). Each column represents the cumulative number of patients for a given serotype.

The *S*. *pneumoniae* serotype distribution was analyzed within the NP samples of the five cohorts with the 40-PCR assay (Fig 1A). The major serotypes or serogroup found in the French cohort were 7A/F (23.3%), 1 (23.3%), 3 (18.6%), 35F/47F (11.6%), and Sg6 (9.3%). The Brazilian cohort had a predominance of serotype 14 (30.8%) and serogroup Sg6 (21.2%). In the South African cohort, 38 serotypes among the 40 included in the 40-PCR assay were identified; the most highly-represented serotypes or serogroup were 3 (13.8%), Sg6 (12.2%), and 19F (11%). In the Malian cohort, the major serogroup was Sg6 (30.7%), followed by serotypes 9A/V (12%) and 1, 19A and 19F (all < 10%). In the Cambodian cohort, the major serogroup was Sg6 (48.4%), followed by serotypes 19F (19.5%), 19A (14.8%), and 23F (10.2%).

Serotype distribution was analyzed in the WB samples with the 40-PCR assay (Fig 1B). In the French cohort, three serotypes [7A/F (66.7%), 1 (33.3%) and 35F/47F (33.3%)] were found in the WB samples. Serotype 1 was the major serotype found in the WB samples of the South African cohort (19.6%) and Malian cohort (50%). Serotypes 3 (15.7%) and 19A (13.7%) were only found in the WB samples of the South African cohort. Serotypes 35F/47F (25%) was only found in WB samples from the Malian cohort.

The 81 WB samples from patients in South Africa have been serotyped using the Quellung method and only 9 out of 81 gave a positive serotyping result. For these samples, concordance with the results of the 40-PCR assay was observed in 8/9 (89%) of cases. The serotype of the case not detected by the 40-PCR assay was identified as serotype 17B by the Quellung serotyping test.

### Pneumococcal serotype-specific quantification

Quantification of bacterial load was performed on a total of 178 pneumonia patients from the South African cohort of which both NP and WB samples were available and all of them were tested for *S*. *pneumoniae*. A total of 178 NP samples and 50 WB were *S*. *pneumoniae*-positive samples with at least one identified serotype. The 50 WB *S*. *pneumoniae*-serotypes were considered as invasive serotypes in the corresponding NP samples. Among these 50 samples, 36 were available for quantification. One hundred and twenty one out the 128 remaining samples with non-invasive serotypes were found with one (n = 101), two (n = 16) or three (n = 4) serotypes with a total of 145 different serotypes.

For all patients for which both NP and WB samples were available, the same serotype was detected in both samples. For each case where multiple serotypes were present in the NP sample, the serotype with the highest bacterial load was the serotype detected in the WB sample.

**Fig 2:**
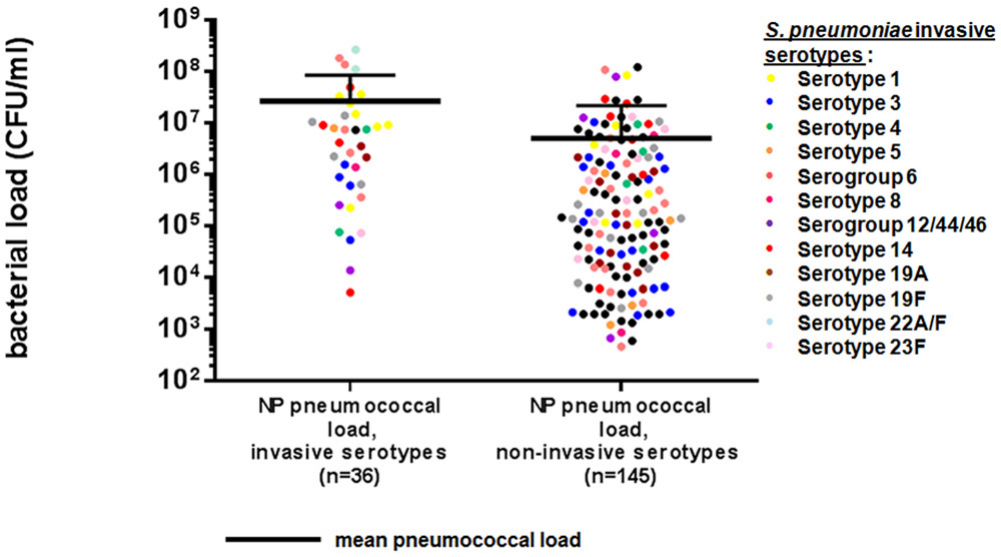
Pneumococcal load distribution in NP samples from the South African cohort. Comparison between invasive (present in both NP and WB samples of the same patient; n = 36) and non-invasive serotypes (present in NP samples only; n = 145). Each plot represents a sample. The bold bars indicate the mean bacterial concentration for each group (Student's *t*-test, *P*<0.001).

The mean concentration of invasive serotypes (i.e. serotypes found in both the NP and WB samples of the same patient, n = 36) in NP samples was higher than the concentration of non-invasive serotypes in NP samples (Fig 2; respectively, 263.4 x 10^5^ CFU/ml [± 570.7 x 10^5^ CFU/ml] vs. 49.9 x 10^5^ CFU/ml [±167.4 x 10^5^ CFU/ml], *P* < 0.001). The median concentration of invasive serotypes in NP samples was 57.0 x 10^5^ CFU/ml (IQR: 6.2 to 143.2 x 10^5^ CFU/ml) compared to 1.46 x 10^5^ CFU/ml (IQR: 0.16 to 21.5 x 10^5^ CFU/ml) for non-invasive serotypes. Moreover, the range of serotype concentrations was larger in NP samples (5.2 x 10^3^ to 2.6 x 10^8^ CFU/ml) than WB samples (1.6 x 10^2^ to 3.5 x 10^5^ CFU/ml). In patients with invasive serotypes (n = 36), the median concentration of the invasive serotype in NP samples was 50.0 x 10^5^ CFU/ml (IQR: 6.2 to 143.2 x 10^5^ CFU/ml) compared with 0.05 x 10^5^ CFU/ml (IQR: 0.02 to 0.16 x 10^5^ CFU/ml) for the concentration of the same serotype in WB samples (*P* = 0.007).

## Discussion

*Streptococcus pneumoniae* is the most prevalent pathogen implicated in CAP in children and adults. Several techniques for serotyping pneumococci have been described. The current reference standard is the Quellung reaction (also called Neufeld’s swelling reaction); however, only a small number of (inter)national reference laboratories have the necessary expertise to perform and interpret this assay. Alternative methods include the latex agglutination assay [13], ELISA [29], colony blot [30], dot blot [31], immunoblot [32], microarray [33] or microsphere-based liquid arrays [34]. All of these methods require previous culture of the pneumococci and thus do not provide information regarding the serotype-specific load of the original sample. Molecular approaches for serotyping such as multiplex PCR assays have already been described; however, these methods are either non-quantitative as they are based on traditional PCR [23] or can only detect a limited number of serotypes [35]. An inexpensive typing method for *S*. *pneumoniae* (estimated at less than 10€ per sample) that can directly use clinical samples -without the need for culture- does not exist and would be highly desirable.

Here, we propose a novel alternative method based on quantitative multiplex real-time PCR for the 40 most prevalent serotypes of *S*. *pneumoniae*. The novel quantitative multiplex real-time PCR method is based on Taqman probe chemistry and is highly specific. Based on strains tested and sequences alignment, the current methodology cannot distinguish serotypes within some serogroups (6A/B/D/E, 7B/C, 7A/F, 9A/V, 9N/L, 10C/F, 11A/D, 12A/B/F, 15A/F, 15B/C, 18A/B/C/F, 22A/F, 24A/B/F, 33A/F, 35A/C), which can be explained by the difficulty of designing specific primers due to the high similarity of the *cps* locus among members of the same serogroup[36,37]. Moreover, a small number of serotypes cross react with serotypes belonging to a different serogroup (serotype 38/serogroup 25, serogroup 12/serotypes 44 and 46, serotypes 33A/F/serotype 37, serotypes 35A/C/serotype 42, serotype 35F/serotype 47F and serotypes 7B/C/serotype 40). The high genetic similarity within the *cps* gene clusters between different serotypes and/or serogroup explain the cross reactions we observed [37–40]. However, serotypes 10C, 33D, 40, 42, 44 and 47F are not encountered serotypes and recent studies of *S*. *pneumoniae* serotypes surveillance did not shown that this serotypes are circulating [41–45]. The typing assay is only performed on *S*. *pneumoniae-*positive samples (*lytA* PCR assay) and non-specific detection of related bacterial species likely to be present in clinical samples (*S*. *mitis*, *S*. *oralis*, *S*. *pseudopneumoniae*, *S*. *sanguinis*, *S*. *parasanguinis*, *S*. *pyogenes*, *S*. *agalactiae*) was not observed in any sample.

The analytical sensitivity depends on the serotype. The detection limit of the assay was below 100 CFU/reaction for 91% of the serotypes. These serotype-dependent variations in assay sensitivity are most certainly due to primer-probe interactions in the multiplex PCR, or varied DNA extraction efficacy that may potentially be related to the capsule composition. The assay was optimized in order to find an acceptable compromise between the number of serotypes targeted by each multiplex reaction and the sensitivity of the assay.

NP (*n =* 562) and WB (*n =* 102) samples from different cohorts of patients from five countries (South Africa, Cambodia, Brazil, Mali, France) representing four continents were used to evaluate the typing assay. More than one serotype was found in 56.2% of the NP samples, which reflects the carriage of multiple *S*. *pneumoniae* strains in the respiratory tract. In the blood, potentially more than one serotype was detected in 24.6% of WB samples. In some cases, the assay is not able to determine the exact number of serotypes because of serogroups detection or cross-reactions. For a vast majority of patients (82.4%) for which a serotype is found in the WB sample, the same serotype is found in the corresponding NP sample.

The 40-PCR assay enables identification of the 40 main *S*. *pneumoniae* serotypes, and identified the serotypes present in 89% of the NP samples overall, ranging from 85% to 98% depending on the cohort tested. We observed that the sensitivity of the assay was lower in WB (60% to 75% depending on the cohort) than NP samples. This can be explained by the potentially inhibitory effect of blood components in the PCR[46]. The failure of serotype identification in these NP and WB samples was not due to a lack of sensitivity of the assay, since the *lytA* gene was detected. The possibility of cross-reaction with other closely-related species expressing a *lytA* gene was excluded (data not shown). The failure of serotype identification for selected samples is probably due to the fact that the serotypes present in these cases are not included in the major 40 serotypes panel detected by the assay.

Compared to the 4 others cohorts, the South African cohort had a positive PCR result for 38 out of 40 targeted serotypes in the NP specimens, with no major predominant serotype. This is probably due to the fact that this cohort was composed of adults and mostly HIV + hospitalized for pneumonia, whereas the four other cohorts were children aged less than 5-years-old. In the WB samples, the most represented serotype in the French cohort was serotype 7F, whereas serotype 1 was the major serotype in the South Africa and Malian cohorts from the African continent. The WB samples from the South African cohort were previously characterized using the Quellung typing test, which is the reference method for typing *S*. *pneumoniae*, but only nine gave a positive typing result. The typing of 89% of these cases were concordant with the results of the 40-PCR assay. Only one serotype, serotype 17B, was not identified in the 40-PCR assay, as this serotype is not included in the molecular typing test.

The assay was also designed to quantify each serotype independently. If a serotype was identified, the sample was re-analyzed using the corresponding primer-probe set in parallel with serially-diluted DNA prepared from cultures of the same serotype of known bacterial concentration, in order to determine the serotype load in the sample (expressed in CFU/ml). The bacterial serotype concentrations were compared in the NP and WB samples from the South African cohort. The bacterial load was significantly higher in NP samples than WB samples. Interestingly, the serotypes with the highest bacterial loads in the NP samples were also the serotypes identified in the WB samples, which clearly indicates a relationship between the density of colonization and the invasiveness of some serotypes. Moreover, we compared the *S*. *pneumoniae* serotype concentrations in the NP samples of cases where the same serotype was also found in the corresponding WB sample with those of cases where *S*. *pneumoniae* remained only as carriage. The *S*. *pneumoniae* concentrations of individual serotypes in NP were significantly higher in cases where the same *S*. *pneumoniae* serotype was found both in the NP and WB samples. This clearly shows that the invasiveness of *S*. *pneumoniae* serotypes is linked to a high *S*. *pneumoniae* load in the nasopharynx. Several serotypes may co-exist in the respiratory tract; however, usually only one serotype will have the capacity to grow in the deeper regions of the lungs and infiltrate the blood compartment. Therefore, the quantitative typing PCR assay may be a useful tool to predict the invasiveness of specific *S*. *pneumoniae* serotypes in individual patients; this feature would be of significant clinical interest.

Over the past five years, pneumococcal conjugate vaccine (PCV) have been rolled out in emerging and developing countries, where disease burden is the highest, pneumococcal carriage is more intense and the range of serotypes is more diverse [47–49]

It is very crucial to develop means for monitoring PCV impact, and likely serotype replacement. Moreover, accurate monitoring of the *S*. *pneumoniae* serotypes circulating worldwide provides important information for the antigen composition of future vaccines. However, vaccination information was not available for most of the patients in this study; therefore, it was not possible to relate the invasiveness and bacterial load of specific serotypes to the vaccination status of the patient.

In conclusion, we have demonstrated the ability of a novel real-time multiplex PCR assay to detect and quantify 22 *S*. *pneumoniae* single serotypes, 3 *S*.*pneumoniae* serogroups, 9 couple of serotypes belonging to the same serogroup and 6 couple of serotypes or serogroups belonging to different serogroups, which has significant potential for surveillance of pneumococci worldwide. This methodology is convenient and cost-effective to assess the distribution and circulation of pneumococcal serotypes, adapted for developing and emerging countries. The assay can provide results within a few hours without the need for prior bacterial culture, which considerably reduces the risk of contamination between samples. In less than 6 hours, the typing and quantification results are obtained including the extraction step and it takes less than 3h to have the typing results only. Characterization of the carriage of multiple *S*. *pneumoniae* serotypes and surveillance of pneumococcal disease are critical in order to predict and monitor conjugate vaccine effectiveness, measure changes in disease epidemiology and assess the impact of serotype replacement [10]. This typing method described in this study can be performed directly using low volumes of clinical specimens and has the potential to overcome the difficulties associated with traditional culture-based serologic testing methods, especially in terms of throughput and time savings. As little as 200 μl of clinical sample was required to perform the assay which is a real advantage for pediatric patients. Similar molecular tools are now widely used for epidemiological purposes to improve identification of other pathogens and in the epidemiological study of pediatric parapneumonic empyema[50]. For the purpose of this study, we limited the assay to detection of the 40 major serotypes worldwide; the test could be easily upgraded to detect additional serotypes. The assay was validated on different types of *S*. *pneumoniae*-positive clinical samples, such as nasal swabs, washes or aspirates and WB, obtained directly from *S*. *pneumoniae*-positive cases. The assay can also be applied on other clinical sample types, such as pleural effusions or urine for surveillance of pneumonia (data not shown) or cerebrospinal fluid for diagnosis of bacterial meningitis (data not shown). The advantages and potential applications of this method warrant further assessment and validation in large scale clinical studies.
